# Estimating epidemiological data of Multiple sclerosis using hospitalized data in Shandong Province, China

**DOI:** 10.1186/s13023-016-0457-4

**Published:** 2016-06-04

**Authors:** Xiao Liu, Yazhou Cui, Jinxiang Han

**Affiliations:** Shandong Medicinal Biotechnology Center, Key Laboratory for Biotech Drugs of the Ministry of Health, Key Laboratory for Rare Disease of Shandong Province, Shandong Academy of Medical Sciences, Ji’nan, Shandong China; Ji’nan University Shandong Academy of Medical Sciences College of Life Science and Medicine, Ji’nan, Shandong China

**Keywords:** Rare diseases, Prevalence, DISMDOII, Multiple sclerosis, DALY, Burden of disease, Shandong Province

## Abstract

**Background:**

Multiple sclerosis (MS) is a rare chronically debilitating disease. There are few reports on the burden of disease of MS and prevalence in China. The authors intended to estimate disease burden and prevalence of MS in Shandong Province using available epidemiologic data.

**Methods:**

Prevalence was calculated using DISMOD II software based on incidence extrapolated from hospitalization data, case fatality and remission rate from literature as input indexes. Disability-adjusted life year (DALY) was computed with epidemiologic indexes estimated by DISMOD II program.

**Results:**

The prevalence of MS was estimated to be 3.7(95 % CI: 1.65–5.8) and 6.7(95 % CI: 2.7–9.56) cases per 100,000 people for males and for females, respectively. The mean age at onset of MS was 36.0(43.0 ± 30.0 years in males and 33.7(43.4 ± 29.7) years in females. Duration of the disease was estimated to be 34.0 (31.6 ± 21.0) years for males and 39.5(34.9 ± 21.8) years for females. The disease burden in disability-adjusted life years was 3316, comprised of 903 (27.2 %) years of life lost (YLL) and 2413 (72.8 %) years lived with disability (YLD).

**Conclusions:**

Our study highlighted that population in Shandong Province had a high prevalence of MS and the patients had a heavy disease burden. It also revealed that the results obtained in this paper would be useful to provide a reference for establishing specific healthcare policies for this rare disease in Shandong Province.

## Background

Multiple sclerosis(MS) is a rare disease, characterized by chronic course with intermittent relapses, usually ending up with a severe debilitation [1]. Therefore, once diagnosed, patients with MS are forced to live with it for the rest of their lives, leading to significant health, social, and economic problems [2, 3].

According to previous studies, the prevalence of MS has a wide variation. The estimated prevalence of MS is 203.4/100,000 in the United Kingdom and 94.7/100,000 in France, respectively [4, 5]. In the United States, the prevalence of MS has been presumed to be approximately 100/100,000 [5]. The prevalence of MS is estimated to be 3.9 per 100,000 in Japan and 3.5 per 100,000 in Korea [6, 7]. According to WHO, there are more than 2.5 million MS patients worldwide, accounting for 6.29 % of the total neurologic diseases and disease burden of MS accounts for 0.1 % of total disease burden of neurologic diseases. It has been estimated that MS generates 2.9 person-years per 100,000 in Korea [8, 9].

Even though MS is also an intractable and chronically debilitating rare disease in China, yet, until now, to the best of our knowledge, no studies on the prevalence and disease burden of MS are available. In addition, data from other countries may not reflect the country-specific situations. The lack of these important epidemiological data has greatly limited the government to develop a health policy for MS. Therefore, estimation of epidemiologic indexes of MS in China is necessary. In this study, the DISMODII was employed to estimate the prevalence and disease burden of MS in Shandong Province, China.

DISMODII is structured by two basic inputs: the construction of population divided by gender and age, and the entire mortality rate for each demographic group. The model assumes a causal relationship between a set of indicators relevant by age and gender: incidence, prevalence, remission, mortality, duration, case fatality, and the relative risk on total mortality [10, 11]. If three of these disease variables (by age groups and gender) are defined, DISMODII uses a set of mathematical equations to derive an internally consistent set of epidemiological parameters including the prediction of the other disease parameters. In this study, incidence, case fatality and remission rate were used as the three inputs.

## Methods

### Estimation of MS incidence in Shandong Province

Currently, China has no MS disease registration system. In this study, we used the hospitalized patients of MS in 2013 to estimate the incidence of MS in Shandong Province. The study was approved by the Shandong Academy of Medical Sciences’ ethics committees. All data used in this study were accessed in accordance with ethical requirements and did not violate the privacy right of individuals. The hospitalization data were obtained from university-affiliated hospitals, and hospitals at provincial or municipal level from 17 cities in Shandong Province in a national pilot project of rare diseases initiated in China. The system contains registration files of hospitalized patients, which include comprehensive records of demographic data, dates of clinical visits, diagnostic codes, details of prescriptions, and examinations. Based on International Classification of Diseases Ten Revision (ICD-10) code, we collected the patients diagnosed with MS from the system and excluded those patients with uncertainty or repeated registrations. The incidence of MS was calculated using the number of incident cases as the numerator and the Shandong population in 2013 as the denominator.

### Case fatality rate

Since case fatality rate is currently unavailable in China, the relevant data of case fatality rate were obtained from published research from Korea [9]. Data from Korea were used as the reference were based on the following considerations: on the one hand, Korea is the neighboring country with the closest racial comparisons to China in Asia; on the other hand previous studies have demonstrated that there is no significant correlation between survival rate and latitude of regions among Asian races and that the pathogenesis characteristics such as disease’s progress and age are of little difference.

### Other data sources

Remission rate refers to a fraction of individuals with a disease recovering to a normal state. Since MS is assumed to be an irreversible disease, the remission rate is deemed to be equal to zero [12]. The age- and gender-specific population of Shandong Province was obtained from the Statistics Office. Data on overall mortality were drawn from Chinese Center for Disease Control and Prevention.

### Data analysis using DISMOD II software

To estimate prevalence and other epidemiologic data, all data were broken down by age and gender. Incidence, the case fatality and the remission rate, along with general mortality and population were entered into DISMODII. The equation calculated numerically using the finite difference, an iterative approximation method. For estimation purposes, the estimators were assumed to follow a Poisson distribution.

### Burden of disease

To estimate the disease burden, we adopted the approach used in the Global Burden of Disease study, which has been used to estimate rare disease burden in China [13]. According to this approach, DALY was the sum of years of life lost (YLL) and years lived with disability (YLD). To calculate the YLD, number of incident cases, disease duration, disability weight and age at onset were entered into formula. The number of fatal cases and the remaining life expectancy were added to the YLL formula for calculation.

$$ \begin{array}{l}YLL={\displaystyle {\sum}_0^1{D}_i}\times {E}_i\hfill \\ {}YLD={\displaystyle {\sum}_0^1{N}_i}\times {I}_i\times {T}_i\times D\hfill \end{array} $$

Where Ei is the average expected years remaining, Di the number of deaths, Ni the population susceptible to MS at each age, I_i_ MS incidence by age group and sex, T disease duration for each age group, and D is disability weight.

In this study, the Coale and Demeny West Level 26 Life Table was adopted to calculate the remaining life expectancy [14]. Regarding to disability weight, no data have been published in China. Therefore, a disability weight value of 0.690 was adopted in this study. This value was used for analysis of burden of disease of MS patients in Korea [9]. The age-weighting and discounting factors used in the GBD study were applied to calculate DALY. In summary, DALY was calculated with a discount rate of 3 %, an age-weighting modulation factor of k = 1 D, and a disability weight of 0.69.

### Statistical analysis

All statistical analyses are carried out using SPSS 20.0 statistical software. The results are expressed as 95 % confidence intervals (95 % CI) for prevalence. Mean ± SD for are used to express continuous variables.

## Results

Based on the hospitalization data, a set of patients diagnosed with MS for the first time in 2013 were obtained, which were used to estimate the incidence of MS. As seen in Table 1, this study demonstrated an increasing incidence of MS with the increase in ages, peaking at ages15–29 years and 30–44 years for female and male patients, respectively. Generally, female patients had an earlier onset and higher incidence than male patients.

**Table 1:** Input variables in DISMOD II modeling for multiple sclerosis in Shandong Province

Age	Input					
	Incidence ^a^(100000)		Case fatality (100000)		Total population ^b^	
	Male	Female	Male	Female	Male	Female
0–4	0.07	0.07	0.012	0.007	2937849	2396888
5–14	0.06	0.09	0.012	0.007	5210843	4528629
15–29	0.12	0.36	62.7	47.6	11096477	10788151
30–44	0.23	0.28	607.6	509.5	11963422	11776850
45–59	0.11	0.13	1312.2	1364.0	10505956	10457193
60–69	0.05	0.05	2046.6	1864.5	3911022	3936225
70–79	0.14	0.08	2257.7	1574.8	2119215	2342429
80+	0.0007	0.0007	1979.9	2062.5	702160	1119410
Total	0.098	0.13	NA	NA	48446944	47345775

^a^Incidence was estimated using the number of hospitalized patients as the numerator and the Shandong Province population in 2013 as the denominator

^b^Shandong population in 2013

In this research, the prevalence of MS was estimated to be 3.7(95 % CI: 1.65–5.8) cases per 100,000 people for males and 6.7(95 % CI: 2.7–9.56) cases per 100,000 people for females (Table 2). Furthermore, the prevalence tended to increase in both men and women with the increase in ages, reaching a peak at age 45–59 years for both sexes. The mean disease duration of MS was 34.0 and 39.5 years in males and females, respectively. It was also estimated that female patients had an average onset age of 33.7 years, which was slightly earlier than the male patients (36 years) (Table 2).

**Table 2:** Modeled incidence (per 100000 population), prevalence (per 100000 population), age on set, death cases and disease duration by age groups and gender

Output										
Age	Incidence		Prevalence		Duration (yr)		Age of onset (yr)		Death cases	
	Male	Female	Male	Female	Male	Female	Male	Female	Male	Female
0–4	0.07	0.07	0.18	0.18	63.7	68.3	2.5	2.5	0	0
5–14	0.06	0.09	0.67	0.8	56.9	61.0	9.5	10.0	0	0
15–29	0.12	0.37	1.6	3.1	42.8	46.3	24.1	25.0	0	0
30–44	0.22	0.27	4.7	8.9	32.1	35.7	36.7	37.2	4	6
45–59	0.12	0.14	6.2	10.4	22.7	26.1	50.2	50.4	8	15
60–69	0.05	0.05	5.8	9.4	14.4	18.0	65.1	65.0	5	7
70–79	0.13	0.08	5.4	8.5	10.5	13.4	75.7	75.0	3	3
80+	0.02	0.02	5.3	7.8	9.8	10.6	82.5	82.5	1	1
Total	0.12	0.2	3.7	6.7	34.0	39.5	36.0	33.7	21	32

**Fig. 1:**
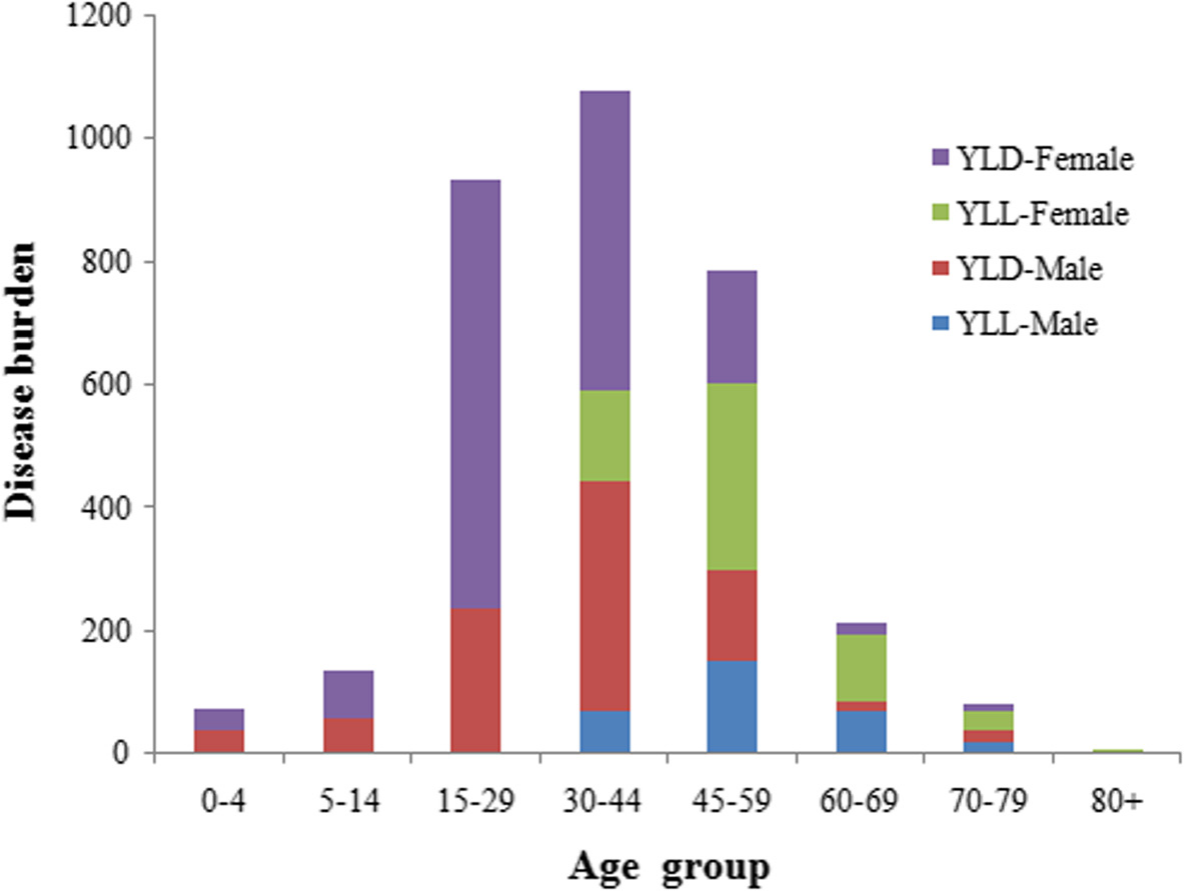
Contribution to DALY by years of life lost (YLL) and years lived with disability (YLD), by age group, in Shandong Province, 2013

The DALY rate of MS in the Shandong population was 3.46 person-years per 100,000. The DALY rate of women (4.46DALYs/100,000) was higher than that in men (2.47DALYs/100,000). Moreover, DALYs in males and females were both the highest in the age of 30–44 years (Table 3). In addition, as shown in Table 3, YLL was 308 for males and 595 for females, whereas YLD was 891 for males and 1515 for females, with a total of 3316. As seen in Fig. 1, it could be seen that different groups of age and gender contributed significantly differently to DALY. With the increase in ages, the percentage of DALY increased due to mortality (YLL). Nevertheless, for patients aged59 years or younger, the most important factor to DALY was morbidity.

**Table 3:** Years of life lost (YLL), years lived with disability (YLD) and disability-adjusted life year (DALY) due to multiple sclerosis in Shandong Province

Age	Male			Female			Total		
	YLL	YLD	DALY	YLL	YLD	DALY	YLL	YLD	DALY
0–4	0	40	40	0	34	34	0	74	74
5–14	0	57	57	0	80	80	0	137	137
15–29	0	236	236	0	697	697	0	933	933
30–44	70	374	444	147	486	633	217	860	1077
45–59	151	149	300	301	185	486	452	334	786
60–69	69	16	85	108	19	127	177	35	212
70–79	18	19	37	32	14	36	50	33	83
80+	0	0	0	7	0	7	0	7	7
Total	308	891	1199	595	1515	2110	903	2413	3316

## Discussion

Although rare disease study is becoming more and more interesting, the exploration of rare diseases epidemiology is still novel in the field [15]. The obtaining of rare disease epidemiological data mainly depends on the disease registration system or extrapolation from other data resources. Currently, there is no specific registration system for rare diseases in China, and the important epidemiological data, especially the prevalence and disease burden of most rare diseases, are lacking. It has been generally acknowledged that such situation represents a major challenge for China to develop its medical policies on rare diseases [16, 17].

The study uses an extrapolation approach based on DISMOD II program to estimate disease burden and prevalence of MS in Shandong Province, China. This strategy has been applied to scleroderma and schizophrenia in the previous studies [18, 19]. Representative epidemiological indexes were input into the DISMOD model, and it was found that both inputs and outputs had almost the same distribution, indicating a sufficient validity and reliability of this strategy. In this study, a duration of disease of 34.0 and 39.5 years for males and females, respectively, which was very well in line with that reported in other countries. For example, the duration of disease is 32.5–49.2 years in Canadianand36 for men and 43 years for women in Norway [20–22], supporting the validity of this study.

A prevalence of MS of 5.2/100,000 people was obtained in Shandong in this study, which was higher than that in Japan (3.9/100,000) and Korea (3.5/100,000), and much lower than that in European countries (203.4/100,000 in United Kingdom, 94.7/100,000 in France) and the United States (100/100,000). It should be noted that the incidence of MS was calculated in this study with the hospitalization data against Shandong population, it is inevitable that those outpatients diagnosed with MS would not be included and some other patients with MS might be hospitalized in other hospitals that were not covered in this study. Therefore, the parameters of MS we used in this study might represent the low limit and the prevalence of MS obtained should be regarded as the lowest prevalence in Shandong Province.

Incidence and prevalence are often used as one measure index of disease frequency since their calculation only concerns the number of cases and the basic population. However, DALYs consider not only survival time but also life quality caused by a certain disease and their calculation requires age and sex dependent incidence, case fatality, severity of disease and life expectancy. Therefore, DALY is a more comprehensive health indicator to demonstrate the threat of some disease to the whole population. In this study, the major contributing factor to DALY was YLD, indicating that most of the burden of disease was from the loss due to disability from the disease. This result demonstrated that MS could cause high disease burden since it occurs mainly at childbearing age. The DALY rate was estimated to be3.46 person-years per 100,000 in Shandong, which was slightly higher than that reported in Korea(2.9 person- years per 100,000) [9], therefore, more attention should be paid to MS in Shandong Province with the limited health resources.

In our study, we provide clear evidence that there is a large population of MS patients with a high disease burden in Shandong Province. Despite the lack of specific treatment to cure MS, some orphan drugs have been validated to bring about a notable improvement in quality of life. However, most of the orphan drugs targeting MS are still unavailable in China. We believe that these epidemiologic data obtained in this study will contribute to provide a reference for the development of specific health policies on MS in the future. In addition, these indexes can serve as the basis of epidemiological data for individual disease that can fill in the gap of research on rare diseases whose research has been lagging in China. This evidence-based study on MS will also be useful for promoting industry to research and develop innovative orphan drugs for the treatment of this devastating rare disease.

Although our results are promising, the limitations of the present study should be addressed. The first is that the effects of some factors, such as social and economic status, health services and environmental hygiene, were not quantified precisely. With this regard, the WHO standards, the widely accepted world standards, were adopted in this study. However, there are some discrepancies between the standards and the actual domestic environment. The second is that the data used to calculate incidence are insufficient. However, the reality in China is that rare disease data are divided into different age groups or come with restrictive conditions, making it difficult to use them. In addition, previous study has demonstrated that 92.5 % of rare diseases are diagnosed in university hospitals, provincial hospitals, and municipal hospitals [16]. And China has taken a series of effective measures and attempts to improve the diagnosis of rare diseases [23]. Therefore, the incidence obtained in this study is appropriate for use. In the future, we would collect more MS information to complement this study. We believe when these data become available, we can estimate epidemiological parameters more precisely.

## Conclusion

In this study, using a DISMODII model, we estimated prevalence and disease burden of MS in Shandong Province. Our data indicate that there are a large number of MS patients with high disease burden in Shandong Province, and that the current healthcare policies need to be emphasized on this severe rare disease. The result of this paper also contributes significantly to the international readers because there are no reports available for them on the prevalence and disease burden of MS in China.

## Abbreviations

CDC, Centers for disease prevention and control; CI, confidence intervals; DALY, disability-adjusted life years; MS, multiple sclerosis; YLD, years lived with disability; YLL, years of life lost
